# FKBP51-Hsp90 Interaction-Deficient Mice Exhibit Altered Endocrine Stress Response and Sex Differences Under High-Fat Diet

**DOI:** 10.1007/s12035-023-03627-x

**Published:** 2023-09-19

**Authors:** Lisha Wang, Jakub Wojcieszak, Rajnish Kumar, Zhe Zhao, Xuelian Sun, Shaoxun Xie, Bengt Winblad, Pavel F. Pavlov

**Affiliations:** 1 Department of Neurobiology, Care Sciences and Society, Division of Neurogeriatrics, Karolinska Institutet, 17164 Solna, Sweden; 2 Department of Pharmacodynamics, Medical University of Lodz, 90151 Lodz, Poland; 3 Department of Pharmaceutical Engineering & Technology, Indian Institute of Technology (BHU), 221005 Varanasi, India; 4 Department of Toxicology, School of Public Health, Peking University, 100191 Beijing, China; 5 National Clinical Research Center for Geriatrics and Department of Gerontology and Geriatrics, West China Hospital, Sichuan University, 610041 Chengdu, China; 6 Theme Inflammation and Aging, Karolinska University Hospital, 14186 Huddinge, Sweden

**Keywords:** FKBP51-Hsp90 interaction, Mouse knock-in model, Stress, Metabolism

## Abstract

FK506-binding protein 51 kDa (FKBP51), encoded by *Fkbp5* gene, gained considerable attention as an important regulator of several aspects of human biology including stress response, metabolic dysfunction, inflammation, and age-dependent neurodegeneration. Its catalytic peptidyl-prolyl isomerase (PPIase) activity is mediated by the N-terminal FK506-binding (FK1) domain, whereas the C-terminal tetratricopeptide motif (TPR) domain is responsible for FKBP51 interaction with molecular chaperone heat shock protein 90 (Hsp90). To understand FKBP51-related biology, several mouse models have been created. These include *Fkbp5* complete and conditional knockouts, overexpression, and humanized models. To dissect the role of FKBP51-Hsp90 interaction in FKBP51 biology, we have created an interaction-deficient mouse (*Fkbp5*^*TPRmut*^) by introducing two-point mutations in the TPR domain of FKBP51. FKBP51-Hsp90 interaction-deficient mice are viable, fertile and show Mendelian inheritance. Intracellular association of FKBP51 with Hsp90 is significantly reduced in homozygous mutants compared to wild-type animals. No behavioral differences between genotypes were seen at 2 months of age, however, sex-dependent differences were detected in Y-maze and fear conditioning tests at the age of 12 months. Moreover, we have found a significant reduction in plasma levels of corticosterone and adrenocorticotropic hormone in *Fkbp5*^*TPRmut*^ mice after acute stress. In contrast to *Fkbp5* knockout mice, females of *Fkbp5*^*TPRmut*^ showed increased body weight gain under high-fat diet treatment. Our data confirm the importance of FKBP51-Hsp90 interactions for stress-related endocrine signaling. Also, *Fkbp5*^*TPRmut*^ mice can serve as a useful in vivo tool to discriminate between Hsp90-dependent and independent functions of FKBP51.

**Supplementary Information:**

The online version contains supplementary material available at 10.1007/s12035-023-03627-x.

## Introduction

FK506-binding protein 51 kDa (FKBP51) is one of the co-chaperones of heat shock protein 90 (Hsp90) and is encoded by *Fkbp5* gene. In humans, common single nucleotide polymorphisms (SNPs) in *FKBP5* have been identified interacting synergistically with environmental factors to increase susceptibility to develop stress-related disorders, such as post-traumatic stress disorder (PTSD), depression, and anxiety [1]. In addition, FKBP51 is involved in metabolic regulation. SNPs in *FKBP5* are associated with type 2 diabetes mellitus (T2D) [2], body weight change [3] and insulin resistance [4]. FKBP51 expression in human subcutaneous adipose tissue tends to be increased in T2D subjects and is related to adipogenesis, glucose and lipid metabolism [5]. Therefore, FKBP51 is an important link between stress-related disorders and T2D-related metabolism.

FKBP51 negatively regulates glucocorticoid receptor (GR) by reducing its binding to glucocorticoids (GC), delaying GR nuclear translocation, and thereby decreasing GR-dependent transcriptional activity [6]. Meanwhile, the expression of FKBP51 itself is regulated by GR-induced transcription via GC response elements in *Fkbp5* locus. This completes an ultra-short negative feedback loop on GR sensitivity. In addition to its GR modulatory property, FKBP51 has been shown to interact with other signaling pathways related to glucose and fat metabolism, such as PPARγ signaling and adipogenesis, PI3K/AKT2 in insulin signaling, and Beclin1 autophagy signaling [7].

To explore biological functions of FKBP51, several mouse models have been generated including *Fkbp5* complete and conditional knockouts, overexpression and humanized mouse models (for a recent review see [8]). Of them, *Fkbp5* knockout (KO) mice were the most studied. Compared to wild-type (WT) mice, *Fkbp5* KO mice are resistant to acute stress [9, 10], do not exhibit chronic stress-induced depressive-like behaviors [11], and show reduced hypersensitivity in persistent pain models [12]. In the context of metabolism, *Fkbp5* KO mice are resistant to diet-induced obesity and exhibit elevated glucose and insulin tolerance under high-fat diet (HFD) condition [13, 14].

FKBP51 encompasses three domains: N-terminal FK506-binding domain (FK1) possessing peptidyl-prolyl isomerase (PPIase) activity, a scaffolding FK2 domain without PPIase activity, and a C-terminal tetratricopeptide repeat (TPR) domain responsible for interacting with the Hsp90 C-terminal peptide Met-Glu-Glu-Val-Asp (MEEVD) [15]. Different FKBP51 domains mediate its interactions with specific sets of partner proteins. Inhibitory effect of FKBP51 on the GC signaling is mediated by GR-Hsp90-FKBP51 complex [16], whereas binding of FKBP51 to GSK3β kinase is largely dependent on FK1 domain [17]. Both FK1 and TPR domains contribute to the binding of FKBP51 to AKT1 kinase [18]. Specifically, a cryo-EM structure of GR-Hsp90-FKBP51 complex showed that FKBP51 not only binds to GR indirectly via Hsp90 MEEVD:TPR interface, but also directly via three interfaces, namely FK1:GR, FK2:GR Helix 3 and FK2-TPR linker:GR Helix 12 [19]. Meanwhile, other TPR motif containing co-chaperones, especially FKBP52, can compete with FKBP51 to bind the GR-Hsp90 complex [20].

To differentiate between Hsp90-dependent and independent functions of FKBP51, we generated a novel mouse knock-in model (*Fkbp5*^*TPRmut*^) harboring two-point mutations (K352A, R356A) in the TPR domain of FKBP51 rendering it unable to interact with Hsp90. In this study, we characterized *Fkbp5*^*TPRmut*^ mice behaviors under basal conditions and explored their reactions under acute stress and HFD conditions. The application of this new *Fkbp5*^*TPRmut*^ mouse model can extend current knowledge on in vivo functions of the FKBP51-Hsp90 complex and provide preclinical proof of concept for a novel therapeutic target, i.e., inhibition of FKBP51-Hsp90 interactions.

## Results

### Generation of FKBP51-Hsp90 Interaction-Deficient Knock-In Mice

Interaction between FKBP51 and Hsp90 is well characterized at molecular level [21]. Negatively charged C-terminal -MEEVD peptide of Hsp90 forms multiple hydrophobic and polar interactions with several key amino acid residues (dicarboxylate clamp) of the TPR domain of FKBP51. We have performed the prediction of binding affinity (ΔG) and dissociation constant (K_d_) of the WT human FKBP51 and K352A and R356A mutated human FKBP51 complexed with MEEVD peptide. The results are presented in the Supplementary Table 1 and Supplementary Fig. 1. The mutated FKBP51 complex with Hsp90 MEEVD peptide showed the loss of the formation of dicarboxylate clamp responsible for anchoring of MEEVD peptide to the TPR domain of the FKBP51. Moreover, the predicted binding affinity (**ΔG**) for the WT and mutated FKBP51-MEEVD complex was − 6.9 and − 5.7 kcal mol^−1^, respectively and dissociation constant (**K**_**d**_) was 8.8 e^−06^ M and 7.0 e^−05^ M, respectively. Indeed, previous reports suggest that the mutation of Lys352 and Arg356 of FKBP51 would completely abolish its interaction with Hsp90 [22, 23].

**Fig. 1:**
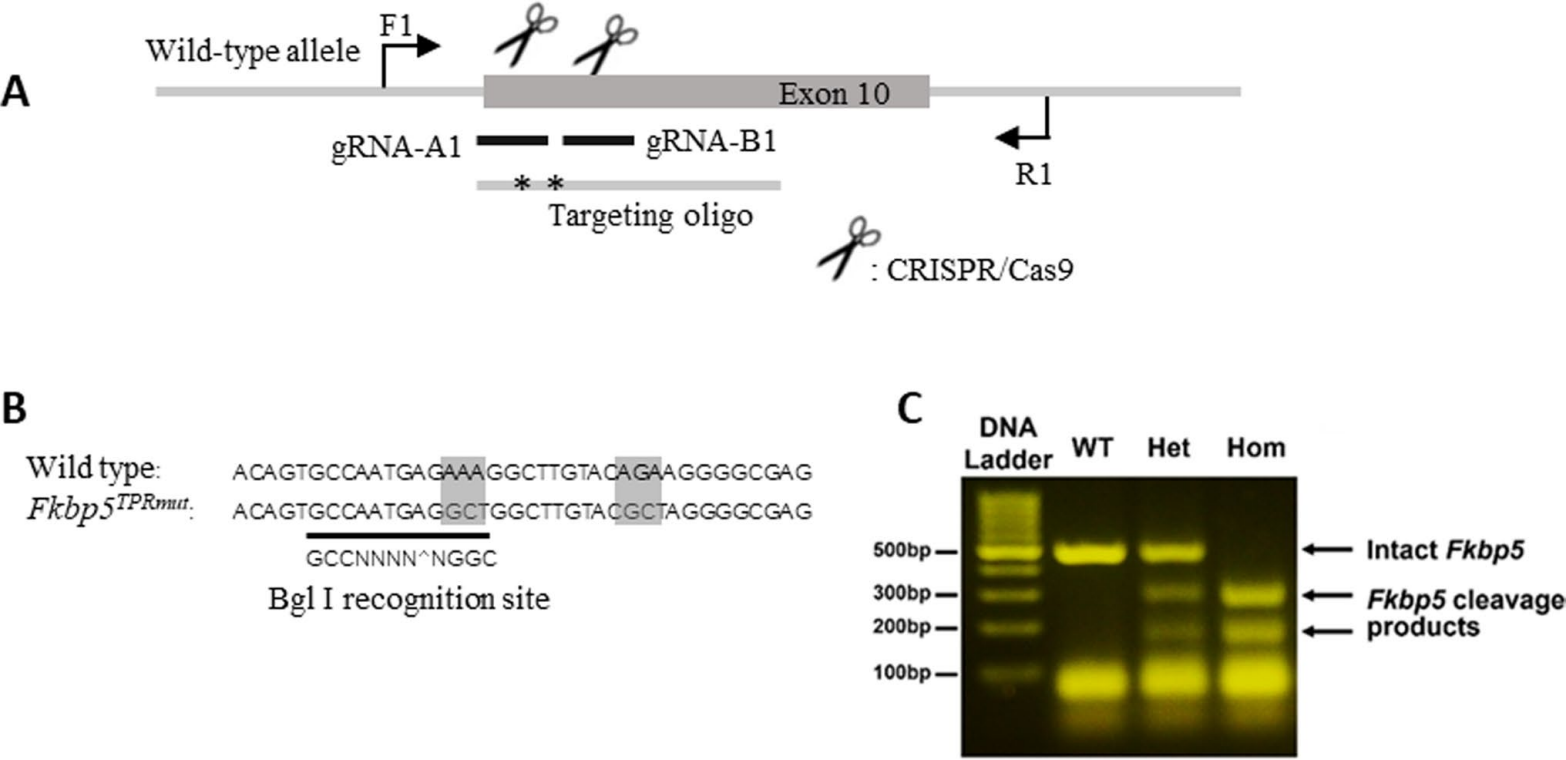
**A** Schematic view of CRISPR/Cas9-mediated gene editing. Mutations (*-*) carried in the targeting oligo were introduced into the wild-type allele of *FKBP5* exon 10 via homology-directed repair using gRNAs and CRISPR/Cas9. Forward (F1) and reverse (R1) sequencing sites are also indicated. **B** DNA sequences flanking mutation sites (marked in shadow). Bgl I recognition site of the mutant allele is underlined. **C** Agarose gel electrophoresis image of the PCR products from wild-type (WT), *Fkbp5*^*TPRmut*^ heterozygous (Het), and *Fkbp5*^*TPRmut*^ homozygous (Hom) mice cleaved with Bgl I restriction enzyme. WT mice displayed one band of intact PCR product at approx. 500 bp, homozygous KO mice displayed two bands at approx. 200 and 300 bp resulting from cleavage products, while heterozygotes displayed all three bands

The mouse *Fkbp5* gene (GenBank accession number: NM_010220.4) is located on chromosome 17 and Lys352 and Arg356 in the TPR domain are encoded within exon 10. CRISPR/Cas9 genome-mediated engineering was used to create K352A and R356A point mutations as described in Materials and Methods. C57BL/6 N mouse strain was used as a background. The targeting strategy and genotyping results are presented in Fig. 1. The gRNA to mouse *Fkbp5* gene, the donor oligonucleotide containing p.K352A (AAA to GCT) and p.R356A (AGA to GCT) mutations site, and Cas9 were co-injected into fertilized mouse eggs that were implanted into surrogate mothers to generate targeted knock-in founders. F0 founder was identified and crossed with the parental strain to produce F1 heterozygotes. When inter-crossed, F1 parents produced WT, *Fkbp5*^*TPRmut*^ heterozygous and *Fkbp5*^*TPRmut*^ homozygous offspring according to Mendelian 1:2:1 ratio.

### Lack of Interactions Between Hsp90 and FKBP51 in *Fkbp5*^*TPRmut*^ Mice

**Fig. 2:**
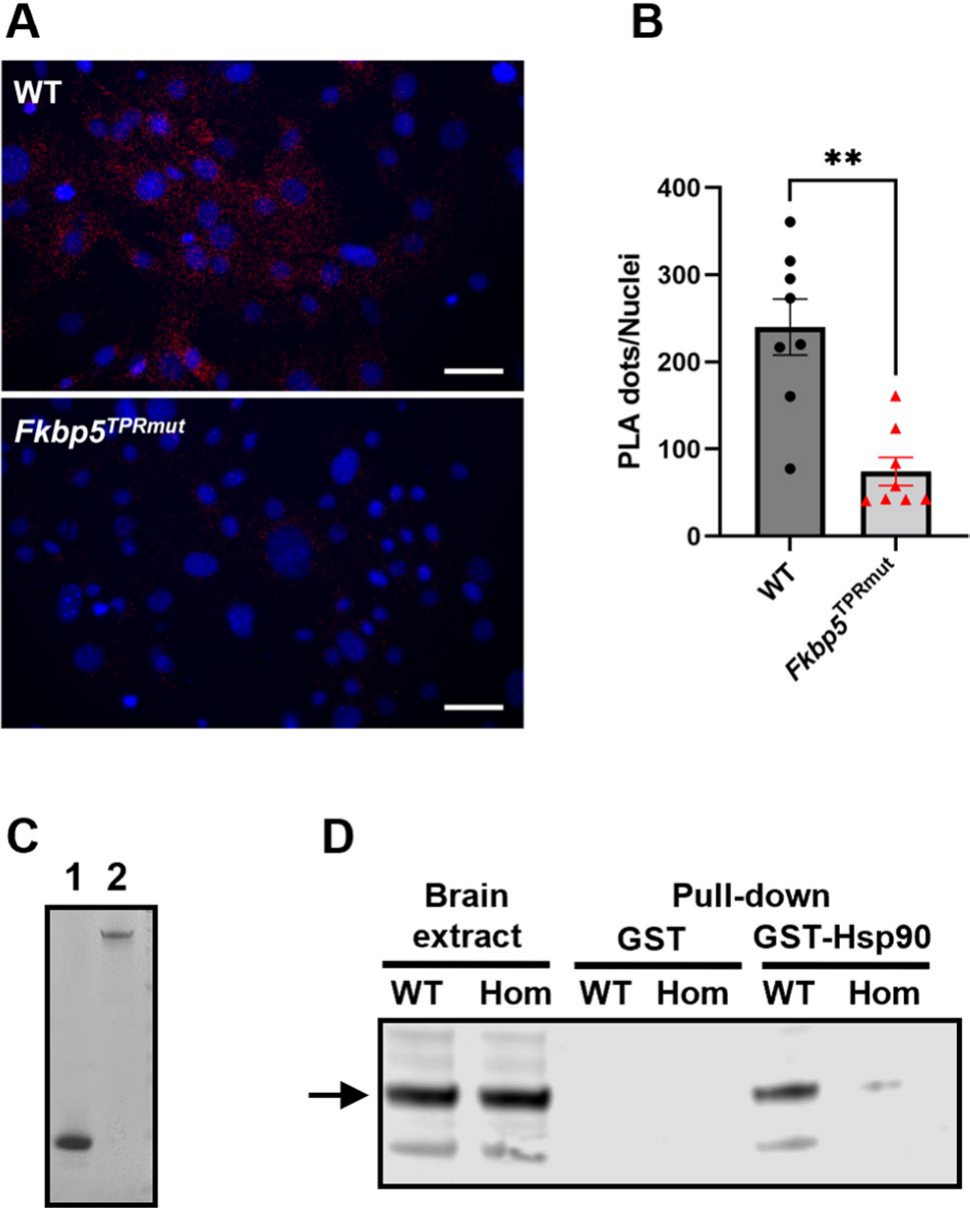
Lack of interactions between Hsp90 and FKBP51 in *Fkbp5*^*TPRmut*^ mice. **A** Images of primary mouse embryonic fibroblasts (MEFs) from wild-type (WT) and *Fkbp5*^*TPRmut*^ homozygous (Hom) embryos, taken by Zeiss Cell observer inverted microscope (40×). Scale bars, 50 μm. **B** Quantification of the PLA signal/Nuclei in WT and *Fkbp5*^*TPRmut*^ groups. Values represent mean ± SEM. ^**^p < 0.01, Mann-Whitney *U* test (two-tailed). **C** Coomassie-stained SDS-PAGE showing isolated GST (lane 1) and GST-Hsp90 (lane 2). **D** Western blot with anti-FKBP51 antibodies. Arrow indicates position of FKBP51

First, we have evaluated the effect of mutations on Hsp90-FKBP51 interactions in cells and tissues isolated from WT and *Fkbp5*^*TPRmut*^ homozygous animals. We performed in situ proximity-ligation assay (PLA) in primary mouse embryonic fibroblasts (MEFs). Using specific anti-FKBP51 and anti-Hsp90 antibodies we have observed a significantly reduced number of dots in MEFs isolated from *Fkbp5*^*TPRmut*^ homozygous embryos than that from WT embryos (Fig. 2A, B) indicating impairment of Hsp90-FKBP51 interactions in the *Fkbp5*^*TPRmut*^ mice. To further corroborate these results, we have performed glutathione-S transferase (GST) pull-down experiments using overexpressed GST-Hsp90 incubated with brain extract from WT or *Fkbp5*^*TPRmut*^ homozygous animals (Fig. 2C, D). Using FKBP51-specific antibodies we detected FKBP51 in the WT brain extract pulled down with GST-Hsp90, whereas much less FKBP51 signal was detected from *Fkbp5*^*TPRmut*^ brain extract. Untagged GST was used as a negative control for these experiments (Fig. 2C, D).

### Behavioral Evaluation and Body Weight Assessment of *Fkbp5*^*TPRmut*^ Mice at Different Ages

**Fig. 3:**
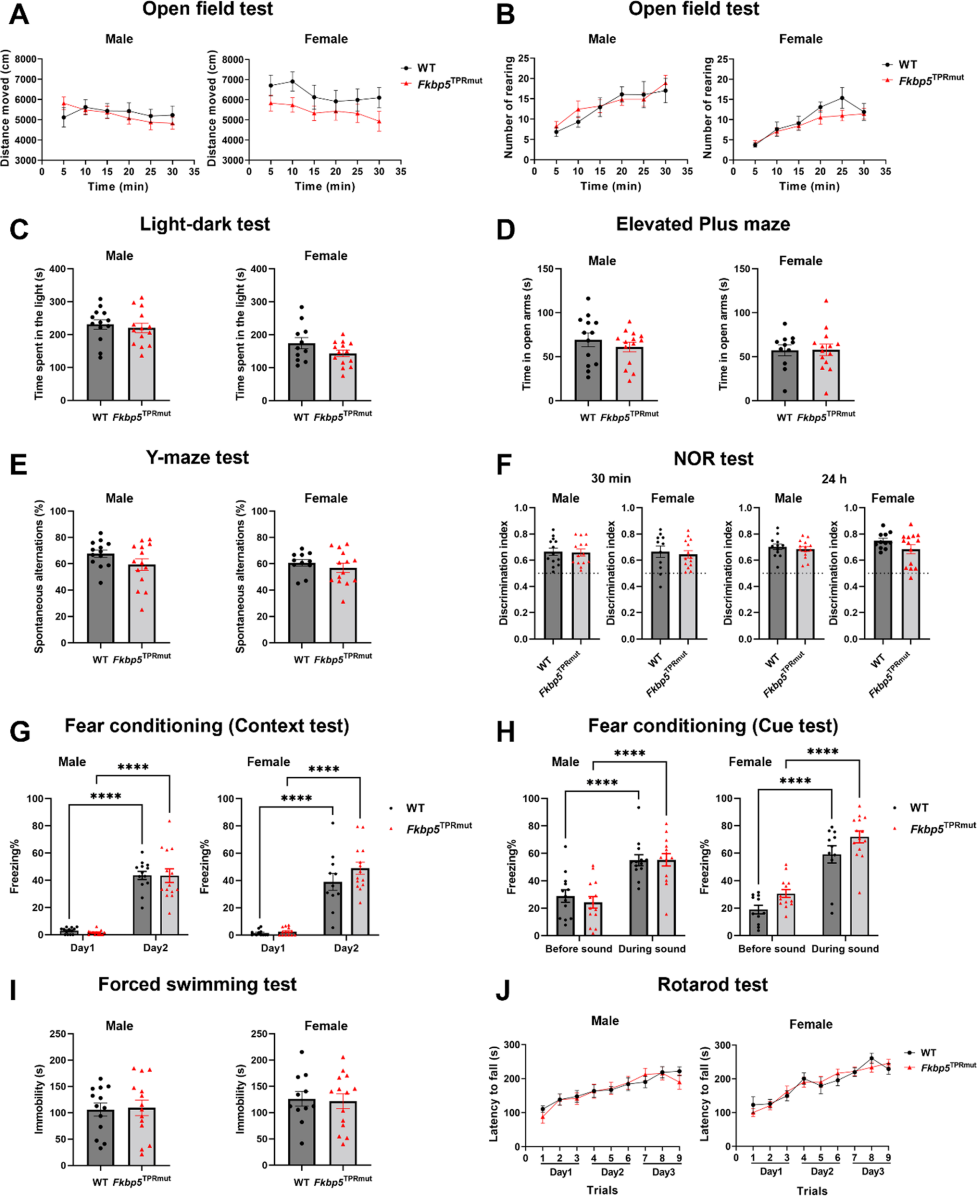
No behavioral impairments in *Fkbp5*^*TPRmut*^ mice at 2 months of age. A battery of behavioral tests was conducted. Distance moved (**A**) and the number of rearings (**B**) in the open field test. **C** Time spent in the light compartment in light-dark test. **D** Time spent in the open arms in elevated plus maze test. **E** Percentage of spontaneous alternations in the Y-maze test. **F** Discrimination index in the novel object recognition test with 30-min or 24-h interval. Percentage of freezing time in the context (**G**) and cue (**H**) tests in fear conditioning. **I** immobility time in forced swimming test. **J** Latency to fall in rotarod test. Values represent mean ± SEM (*n* = 11–14). There are no statistically significant differences between genotypes. **A**, **B**, **G**, **H** and **J**, two-way ANOVA followed by Sidak’s multiple comparison test, ^****^p < 0.0001. **C**, **D**, **E**, **F** and **I**, unpaired Student’s *t* test (two-tailed)

At 2 months of age, a battery of behavioral tests was conducted (Supplementary Fig. 2). *Fkbp5*^*TPRmut*^ mice exhibited similar behaviors as WT mice, both in males and in females (Fig. 3), including spontaneous horizontal or rearing locomotor activity in open field test, anxiety-like behaviors in light-dark and elevated plus maze tests, spatial working memory in Y-maze test, non-spatial object recognition memory in novel object recognition test, fear associated context- and cue-based memory in fear conditioning test, depressive-like behavior in forced swimming test and motor coordination in rotarod test.

**Fig. 4:**
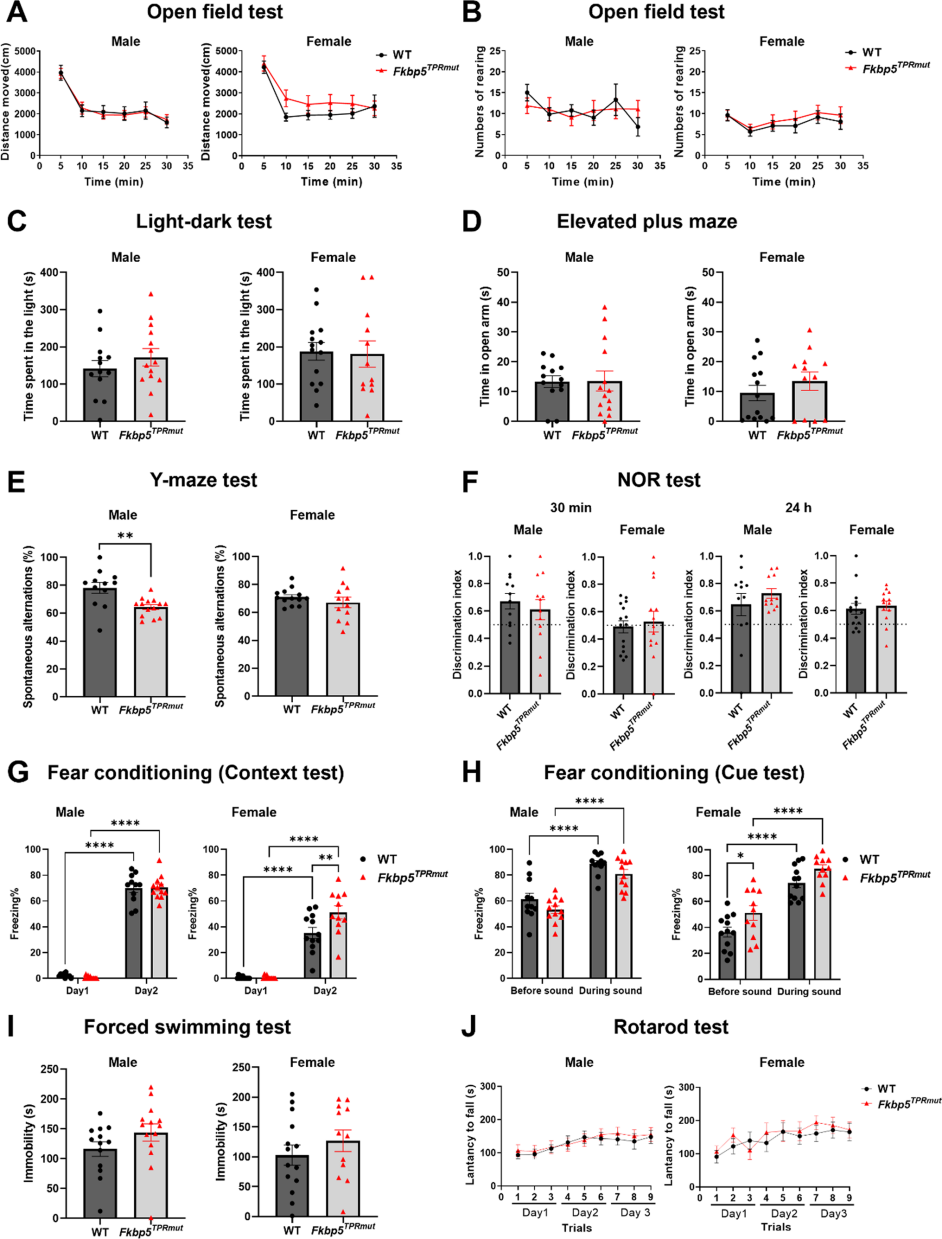
Behavioral assessment of *Fkbp5*^*TPRmut*^ mice at 12 months of age. Distance moved (**A**) and the number of rearings (**B**) in the open field test. **C** Time spent in the light compartment in light-dark test. **D** Time spent in the open arms in elevated plus maze test. **E** Percentage of spontaneous alternations in the Y-maze test. **F** Discrimination index in the novel object recognition test with 30-min or 24-h interval. Percentage of freezing time in the context (**G**) and cue (**H**) tests in fear conditioning. **I** immobility time in forced swimming test. **J** Latency to fall in rotarod test. Values represent mean ± SEM (n = 11–14). **A**, **B**, **G**, **H **and **J**, two-way ANOVA followed by Sidak’s multiple comparison test, ^*^p < 0.05, ^**^p < 0.01, ^****^p < 0.0001. **C**, **D**, **E**, **F** and **I**, unpaired Student’s *t* test (two-tailed), ^*^p < 0.05, ^**^p < 0.01

The same battery of behavioral tests was conducted at 12 months of age (Fig. 4). At this age we have detected sex-dependent differences in Y-maze and fear conditioning tests. *Fkbp5*^*TPRmut*^ males exhibited less spontaneous alternations in Y-maze test compared to WT. In the fear conditioning test, *Fkbp5*^*TPRmut*^ females showed significantly elevated freezing time on Day2 compared to their WT counterparts, indicating they have better contextual fear memory. It has to be noted that the similar trend was observed in 2-month-old animals although values did not reach statistical significance. Overall, these data indicate temporal and sex-dependent abnormalities of *Fkbp5*^*TPRmut*^ mice in learning and memory.

**Fig. 5:**
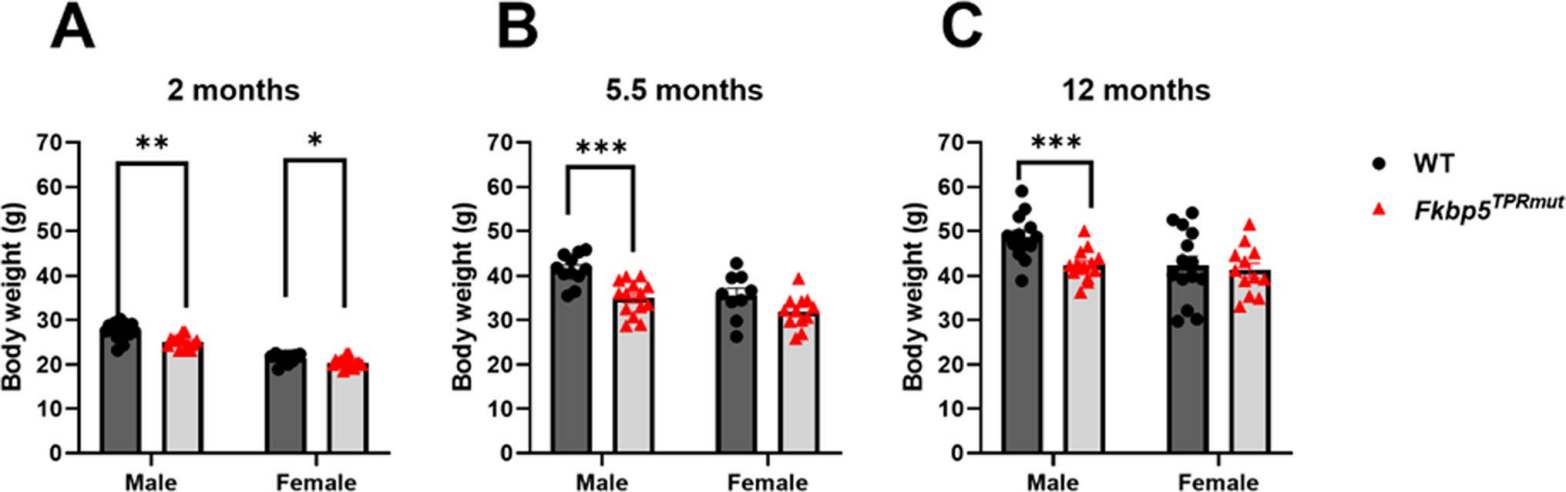
Body weight assessment of WT and *Fkbp5*^*TPRmut*^ mice at various ages. **A** 2 months old, **B** 5,5 months old, **C** 12 months old. Values represent mean ± SEM (*n* = 9–14). Statistical significance between WT and *Fkbp5*^*TPRmut*^ was determined by using Unpaired Student’s *t* test (two-tailed). *p* < 0.05 was considered statistically significant. ^*^, ^**^ and ^***^ represent *p* < 0.05, *p* < 0.01 and *p* < 0.001, respectively

The results of body weight measurement are presented in Fig. 5. Body weight of *Fkbp5*^*TPRmut*^ males was significantly smaller than WT males at any age, whereas no significant differences in body weight between WT and *Fkbp5*^*TPRmut*^ females have been detected at 5,5 and 12 months of age. Our data indicated sex-dependent differences in body weight between WT and *Fkbp5*^*TPRmut*^ animals.

### Reduced Response to Acute Restraint Stress in *Fkbp5*^*TPRmut*^ Mice

**Fig. 6:**
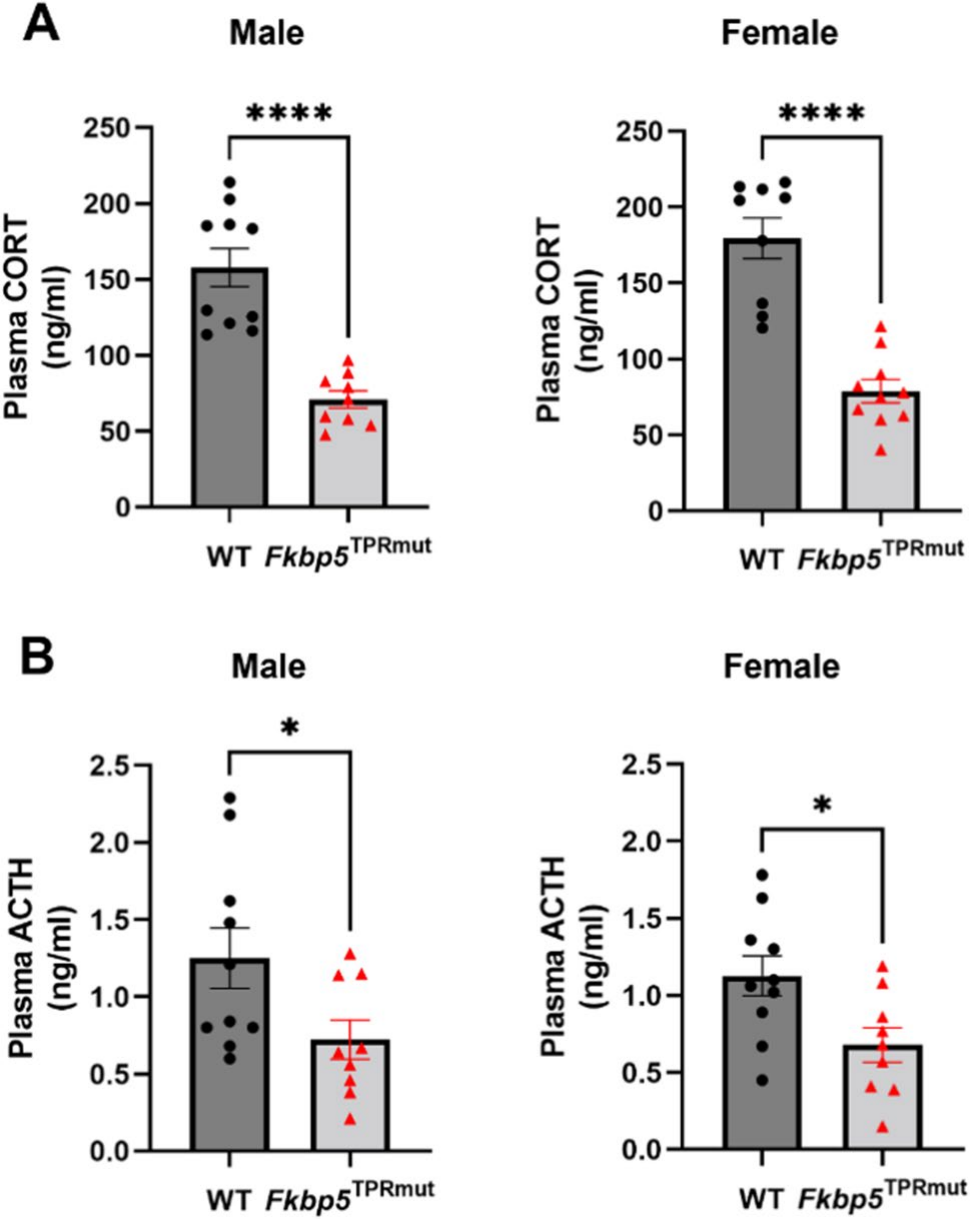
Reduced levels of corticosterone (CORT) and adrenocorticotropic hormone (ACTH) in *Fkbp5*^*TPRmut*^ mice after acute stress. After 15-min restraint stress followed by 30-min recovery, blood was collected by heparin tubes and ELISA was performed to detect plasma CORT levels (**A**) and ACTH levels (**B**) in male and female mice from WT and *Fkbp5*^*TPRmut*^ groups. Values represent mean ± SEM (*n* = 9–10). **A** ^****^p < 0.0001, Mann-Whitney *U* test (two-tailed). **B** ^*^p < 0.05, Unpaired Student’s *t* test (two-tailed)

After 15-min restraint stress followed by 30-min recovery, plasma corticosterone (CORT) and adrenocorticotropic hormone (ACTH) levels were detected by commercial ELISA kits. Results show that the levels of CORT and ACTH in *Fkbp5*^*TPRmut*^ mice were significantly lower than those in WT, both in males and females (Fig. 6). This suggests that *Fkbp5*^*TPRmut*^ mice have reduced response (lower levels of CORT) to acute restraint stress, due to lower circulating levels of ACTH, which is produced by the pituitary gland and stimulates CORT release by the adrenal cortex.

### Deficiency in FKBP51-Hsp90 Interactions Causes GR Hypersensitivity

**Fig. 7:**
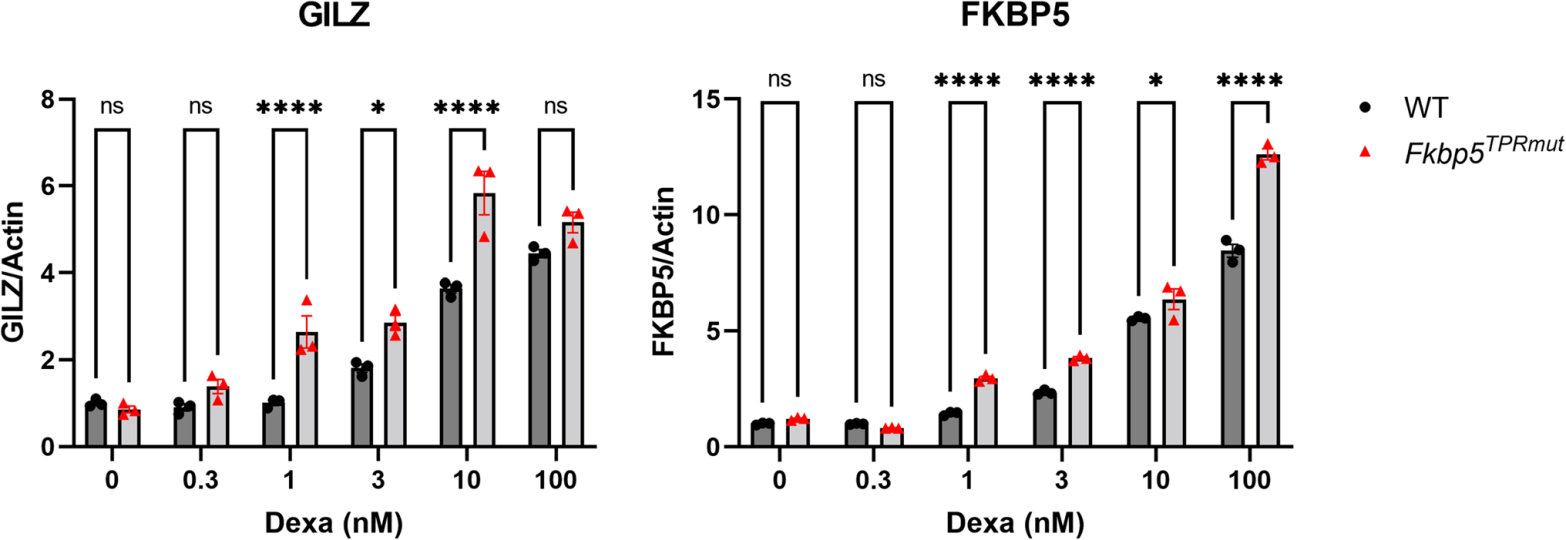
qPCR gene expression measurement in primary mouse embryonic fibroblasts (MEFs) isolated from WT and *Fkbp5*^*TPRmut*^ mice 4 h after dexamethasone (Dexa) treatment. Values represent mean ± SEM (*n* = 3). Statistical significance between groups was tested by two-way ANOVA followed by Sidak’s multiple comparison test. *p* < 0.05 was considered statistically significant. ^*^p < 0.05 and ^****^p < 0.0001

The reduced endocrine response in *Fkbp5*^*TPRmut*^ mice after restraint stress prompted us to investigate the expression profile of GC-responsive genes upon hormonal stimulation. MEF cells were treated with increased concentrations of dexamethasone and, after 4 h, the expressions of GILZ and FKBP51 were measured by the qPCR. The results in Fig. 7 show that hormone-induced expressions of GILZ and FKBP51 were significantly higher in MEFs isolated from *Fkbp5*^*TPRmut*^ mice compared to the WT MEFs.

### Sex Differences in the Body Weight Gain of *Fkbp5*^*TPRmut*^ Mice Under HFD Condition

**Fig. 8:**
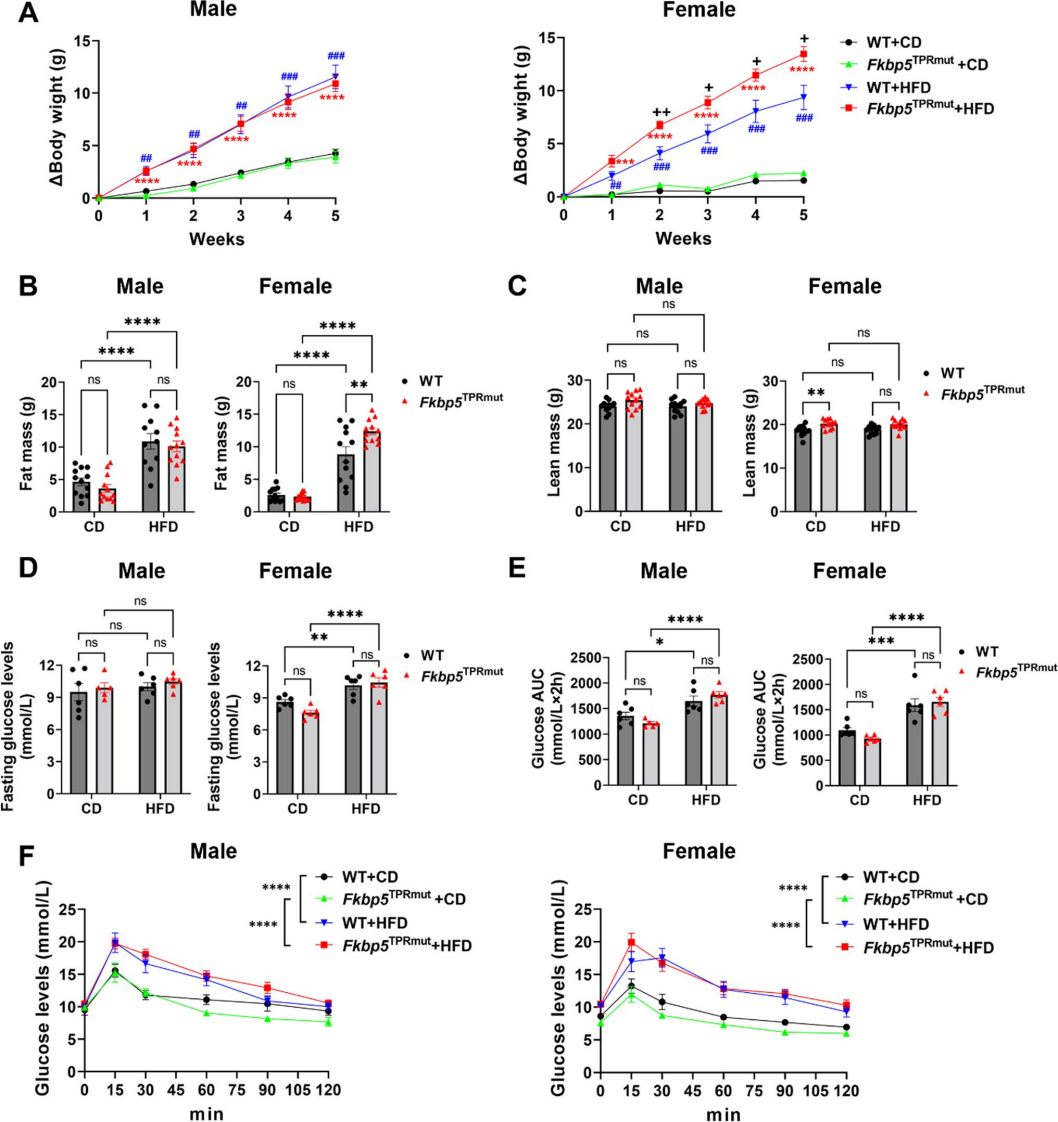
Body weight gain and glucose tolerance in WT and *Fkbp5*^*TPRmut*^ mice under HFD. **A** Body weight gain in WT and *Fkbp5*^*TPRmut*^ mice treated with CD or HFD for 5 weeks. Data are represented as mean ± SEM (*n* = 11–12). The whole-body fat mass (**B**) and lean mass (**C**) measured by EchoMRI-100™ after 5-week treatments. Data are represented as mean ± SEM (*n* = 11–12). Fasting glucose levels (**D**), glucose AUC (**E**) and the curve of glucose tolerance test (**F**) were detected after 5-week treatments. Values represent mean ± SEM (*n* = 5–6). (**A**)-(**F**), Two-way ANOVA followed by Sidak’s multiple comparison test. **A** ^+^*p* < 0.05, ^++^*p* < 0.01, *Fkbp5*^*TPRmut*^ + HFD vs. WT + HFD; ^***^p < 0.001, ^****^p < 0.0001, *Fkbp5*^*TPRmut*^ + HFD vs. *Fkbp5*^*TPRmut*^ + CD; ^##^*p* < 0.01, ^###^*p* < 0.001, WT + HFD vs. WT + CD. **B**-**F**, ns, not significant, ^*^p < 0.05, ^**^p < 0.01, ^***^p < 0.001, ^****^p < 0.0001

At 2 months of age, both male and female WT and *Fkbp5*^*TPRmut*^ mice were treated with control diet (CD) or HFD for 5 weeks. After one-week treatment with different diets, both male and female WT and *Fkbp5*^*TPRmut*^ mice under HFD had higher body weight gain compared to mice under CD (Fig. 8A). This trend lasted until the end of the experiment. In males, there was no statistically significant differences of body weight gain between *Fkbp5*^*TPRmut*^ and WT mice under HFD. However, in females, the body weight of *Fkbp5*^*TPRmut*^ mice increased faster than WT mice, starting after 2-weeks of HFD treatments. At the end of diet treatment, the whole-body fat and lean mass were measured, and glucose metabolism of mice was evaluated by glucose tolerance test (GTT). The higher body weight gain of female *Fkbp5*^*TPRmut*^ under HFD came from higher fat mass (Fig. 8B) instead of lean mass (Fig. 8C). Fasting glucose levels were unchanged in males, but in females, *Fkbp5*^*TPRmut*^ and WT mice under HFD had similarly increased fasting glucose levels compared to mice under CD (Fig. 8D). In GTT, both male and female *Fkbp5*^*TPRmut*^ and WT mice under HFD had lower glucose tolerance compared to mice under CD (Fig. 8E and F). But there was no statistically significant difference between WT and *Fkbp5*^*TPRmut*^ mice under HFD (Fig. 8E and F).

### Genotype-Dependent Differential Composition of Hsp90-TPR Proteins Heterocomplexes Associated with GR and Androgen (AR) Receptors

**Fig. 9:**
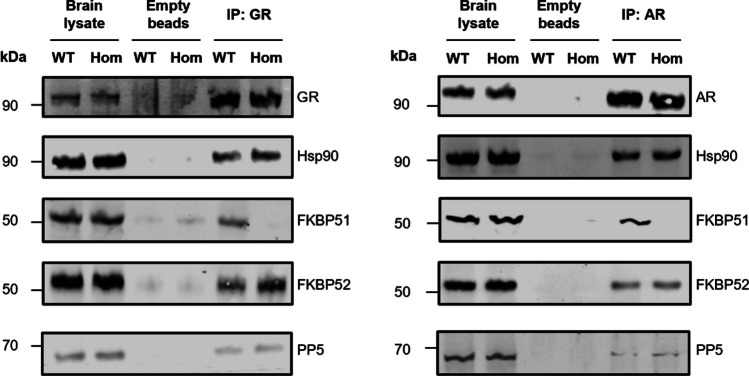
Genotype-dependent differential composition of Hsp90-TPR proteins heterocomplexes associated with glucocorticoid (GR) and androgen (AR) receptors. Whole brains from wild-type (WT) or *Fkbp51*^*TPRmut*^ homozygote mice (Hom) were lysed and incubated with empty agarose beads or with agarose beads coupled to GR and AR antibodies. Protein levels of GR, AR, and associated Hsp90 and TPR co-chaperones were determined by Western blot with respective antibodies

To unravel the molecular impact of FKBP51-Hsp90 interaction deficiency at the levels of nuclear hormone receptors we have investigated the composition of Hsp90-associated TPR co-chaperones co-immunoprecipitated together with GR or AR from adult mouse brains. Whole brain lysates from 12-month-old males were used to pull down protein complexes specifically associated with GR or AR (Fig. 9).

We have probed the immunoprecipitates with specific antibodies against Hsp90, FKBP51, FKBP52 and PP5 which are the major components of GR-associated heterocomplexes. Comparison of GR-associated heterocomplexes isolated from WT or *Fkbp5*^*TPRmut*^ mice brains revealed first: FKBP51-Hsp90 interaction deficiency does not affect total brain levels of studied proteins. Second, FKBP51 from *Fkbp5*^*TPRmut*^ mice is not present among GR-associated proteins. Third, the levels of FKBP52 associated with GR complexes increased in *Fkbp5*^*TPRmut*^ mice as compared to the WT mice, whereas the levels of Hsp90 and PP5 remained unchanged (Fig. 9). Analysis of AR-associated heterocomplexes confirmed the lack of FKBP51 association, however, no differences between genotypes could be seen for FKBP52 binding as well as for Hsp90 and PP5 binding. Therefore, our results support the idea of specific modulation of certain biological functions of FKBP51 via differential regulation of nuclear hormone receptors’ composition.

## Discussion

In this study we report a novel strain of mice (*Fkbp5*^*TPRmut*^) deficient for the interaction between FKBP51 and Hsp90 due to point mutations introduced into the TPR domain of FKBP51. Similar to the complete knockout of FKBP51 and in contrast to deletion of FKBP52, these point mutations within TPR domain of FKBP51 do not affect mice fertility and Mendelian distribution of newborn pups [11, 24]. Like the *Fkbp5* KO mice, *Fkbp5*^*TPRmut*^ mice display reduced sensitivity to the acute stress, as both males and females had significantly lower CORT and ACTH plasma levels in response to 15-min restraint stress, compared to WT mice. This observation is in line with the well-studied role of FKBP51 in the regulation of hypothalamus-pituitary-adrenal axis (HPA). HPA signaling is controlled by the negative feedback loop at several levels. Activation of GR in the brain leads to a rapid decrease of transcription of corticotropin-releasing hormone and ACTH, leading to the reduced release of CORT from adrenals [25, 26]. Lack of interaction of FKBP51 with Hsp90 in *Fkbp5*^*TPRmut*^ mice prevents the formation of a ternary complex with GR, giving functional advantage to FKBP52 via replacing FKBP51 in complex with GR and Hsp90. This results in increased trafficking of GR into the cell nucleus, leading to the enhancement of the negative feedback loop and inhibiting the release of CORT into the blood. We also observed that deficiency in FKBP51-Hsp90 interaction leads to an increased expression of GC-inducible genes including *Fkbp5* upon dexamethasone treatment. Our observations are in line with previously published finding that overexpression of FKBP51 led to increased CORT levels in plasma of rats and mice [27], supporting the positive correlation between functions of FKBP51 and concentrations of CORT in plasma.

We expected that the profound changes in the endocrine response to the acute stress of *Fkbp5*^*TPRmut*^ mice would be reflected in stress-related behavioral tests such as forced swim test, however, no differences between various genotypes or sexes could be detected. Touma and colleagues reported that the additional strong stressors such as prolonged restraint stress are required to observe stress resilience in forced swim test in *Fkbp5* KO males [10]. We conclude that FKBP51-Hsp90 interaction deficiency in *Fkbp5*^*TPRmut*^ mice results in an even milder stress resilient phenotype.

FKBP51 is known to interact with both age and sex. Human and rodent studies demonstrate that FKBP51 expression increases with age [28–30]. In mice, *Fkbp5* deletion prevents the progressive age-associated increases in depression-like behavior [31]. In addition to age, sex is another factor regulating FKBP51 function. Via a negative feedback loop, FKBP51 can inhibit GR signaling that mediates stress-induced sex differences [32, 33]. FKBP51 is also a positive regulator of AR [34, 35], linking its effects to gonadal hormone signaling. In the brain of *Fkbp5*^*TPRmut*^ mice, there is a lack of association between FKBP51 and AR, with no differences of Hsp90, FKBP52 and PP5 binding to AR between genotypes. AR has a beneficial effect on spatial learning and memory [36]. As a positive regulator of AR, FKBP51 stimulates androgen-dependent transcription and cell growth. In *Fkbp5*^*TPRmut*^ mice, FKBP51 could not bind to Hsp90-AR complex, leading to a lower number of AR molecules that undergo androgen binding. This is exacerbated by aging with augmented FKBP51 levels. In addition, male mice have higher AR expression than females in mouse brains [37]. This could explain why we find spatial memory impairment in Y-maze test only in 12-month-old *Fkbp5*^*TPRmut*^ males, not in females or young males. In humans, females have a higher susceptibility to PTSD [38]. Polymorphisms in *FKBP5* have been shown to associate with peritraumatic dissociation, a well-established risk factor for the development of PTSD, in medically injured children [39]. In fear conditioning test, female *Fkbp5*^*TPRmut*^ mice showed longer freezing time during the context test at 12 months of age, indicating that they have stronger contextual fear memory than WT. Interestingly, a recent publication revealed that loss of *Fkbp5* in glutamatergic and GABAergic neurons of older aged mice leads to opposite effects on anxiety only in females and on fear memory only in males [40]. This suggests that FKBP51 modulation is more complex than what has already been known. It needs more targeted and sex-specific studies to reveal a comprehensive role of FKBP51 in the brain.

Higher susceptibility to metabolic stress was observed in females of *Fkbp5*^*TPRmut*^ under HFD. In male mice, global KO of *Fkbp5* gene or pharmacological antagonism of FKBP51 results in increased resistance to HFD effects, including body weight gain, accumulation of adipose tissue, fasting glucose levels and glucose tolerance, without affecting fasting and glucose-induced insulin levels [14]. Varied effects have been reported from studies using manipulations of FKBP51 expression in the hypothalamus. One study reported increased body weight gain and aggravated glucose intolerance in male mice overexpressing FKBP51 in hypothalamus after feeding with HFD, compared to control [27]. Another has reported contrasting results, as deletion of FKBP51 in mediobasal hypothalamus of male mice strongly induced obesity, while its overexpression protected against HFD-induced obesity [41]. Here we found *Fkbp5*^*TPRmut*^ male mice exhibit similar performance as WT under HFD, suggesting that Hsp90-dependent functions of FKBP51 are not involved in the metabolic regulation of male mice. Since there is no reported data on global *Fkbp5* KO females under HFD condition, currently we could not compare our results with *Fkbp5* KO females. A study reported that female AR-KO mice displayed higher increased body weight than control mice under HFD but not under CD [42]. FKBP51 is a positive regulator of AR signaling. The disruption of FKBP51-AR association in *Fkbp5*^*TPRmut*^ mice might mimic the AR-KO effects, which could explain why we find higher body weight gain in female *Fkbp5*^*TPRmut*^ mice under HFD compared to WT. Further research is warranted to determine the exact underlying mechanism.

## Conclusion

In this study we present a novel mouse model, *Fkbp5*^*TPRmut*^, characterized by lack of FKBP51-Hsp90 interaction due to two-point mutations within TPR domain of FKBP51. These mice are viable, fertile and display normal behavioral phenotype under basal conditions. *Fkbp5*^*TPRmut*^ mice are stress-resistant, which makes FKBP51-Hsp90 interaction an interesting target for drug discovery in the field of stress-related diseases. *Fkbp5*^*TPRmut*^ female mice are more sensitive to the effects of fear conditioning and HFD which points to the complexity of the role of FKBP51 as a regulator of stress and metabolism and warrants further research.

## Materials and Methods

### Generation of *Fkbp5*^*TPRmut*^ Mice

*Fkbp5*^*TPRmut*^ mice were generated using GRISPR/Cas9 technology using C57BL/6 N strain as a background and ordered from Cyagen (China). The gRNA to mouse *Fkbp5* gene, the donor oligonucleotide containing K352A (AAA to GCT) and R356A (AGA to GCT) mutations site, and Cas9 were co-injected into fertilized mouse eggs that were implanted into surrogate mothers to generate targeted knock-in founders. The CCTop-CRISPR/Cas9 target online predictor was used to verify that selected gRNA sequences specifically target the *Fkbp5* locus. The selected gRNA sequences produced no off-target modifications in any functional regions of the genome (data not shown). F0 founder mice harboring two-point mutation were identified by PCR followed by DNA sequence analysis and confirmed by high-risk off-targets analysis. F0 founder mice were bred to WT mice strain matched to get the F1 heterozygotes. By mating heterozygous male and female mice, the new generation of WT and *Fkbp5*^*TPRmut*^ homozygous mice was produced. *Fkbp5*^*TPRmut*^ mice were housed with standard conditions at the KM-B animal facility (Comparative Medicine, Karolinska Institutet, Solna, Sweden).

*gRNA target sequences*:

- gRNA-A1 TCTGTACAAGCCTTTCTCATTGG
- gRNA-B1 ATGAGAAAGGCTTGTACAGAAGG

*Donor oligo sequence*:

- CTGTGACCTGCCCACCTGTCCTTCTGTTCTTAGGCCCTTGGACTGGACAGTGCCAATGAG**GCT**GGCTTGTAC**GCT**AGGGGCGAGGCCCAGCTGCTCATGAATGACTTTGAGTCGGCCAAGGGCGACTTCGAGAAG

Mutation sequences are in bold.

### Genotyping

Genomic DNA from the ear biopsy was extracted using DNeasy Blood & Tissue Kits (Cat. No. 6954, Qiagen) following the manufacturer’s instructions. *Fkbp5* was amplified using Phusion High-Fidelity PCR Master Mix (Cat. No. F531L, Thermo Scientific) with *Fkbp5* primers (forward: 5’-TAAAATACTGACAAGTGGGTGGAC-3’, reverse: 5’-CCCTTCCTAAGTCTTATTTCTTCCA-3’). PCR products were separated on 2% agarose gels and sent to KIGene Core Facility (Karolinska Institutet) for DNA sequence analysis. Another method is using restriction enzyme Anza™ 69 Bgl I (Invitrogen) to digest *Fkbp5* mutation site at 37 °C followed by agarose gel electrophoresis.

### PLA

MEFs were isolated from embryos (E13.5) obtained from heterozygous breeding pairs according to a previous protocol [43]. MEFs were cultured in Dulbecco’s modified Eagle’s medium (DMEM, Cat. No. 41965-039, Gibco) with 10% heat-inactivated fetal bovine serum (FBS, Cat. No. 10500-064, Gibco) and 1% Penicillin-streptomycin (Cat. No. 15140-122, Gibco) at 37 °C and 5% CO_2_ with saturated humidity. WT and *Fkbp5*^*TPRmut*^ homozygous MEFs were seeded in the 8-well chamber slide (Cat. No. 177445, Nunc™ Lab-Tek® Chamber Slide™ System) and used for PLA when grown to approximately 80% confluence. PLA was performed using rabbit anti-FKBP51 (Cat. No. GTX66429, GeneTex, dilution ratio 1:300) and mouse anti-Hsp90 antibodies (Cat. No. 37-9400, Invitrogen, dilution ratio 1:300) following manufacturer’s instructions. Images were taken by Zeiss Cell Observer inverted microscope under 40× magnification with same settings. The total cell numbers and the PLA dots were counted by the ImageJ plugin Analyse Particles according to a published method [44]. The number of PLA dots divided by the number of nuclei in each image was calculated to compare the difference of Hsp90-FKBP51 interactions between groups.

### qPCR Assessment of Gene Expression

MEFs from WT or *Fkbp5*^*TPRmut*^ animals were treated either with DMSO (vehicle control) or with  0.3, 1, 3, 10 or 100 nM of dexamethasone for 4 h followed by RNA isolation using RNeasy mini kit (Qiagen). cDNA was synthesized using Maxima First Strand cDNA Synthesis Kit for RT-qPCR with dsDNase (K1671, ThermoFisher Scientific) according to manufacturer’s instructions. The mRNA expression levels of FKBP5 and GILZ were detected by qPCR using Fast Sybr® Green master mix (ThermoFisher Scientific) using applied biosystems 7500 real-time PCR system. Actin was used as an endogenous control to normalize gene expression. Primer pair sequences:

- Actin: 5’-GGCACCACACCTTCTACAATG-3’, 5’-GGGGTGTTGAAGGTCTCAAAC-3’
- FKBP5: 5’-GTGGGTTCTACATCGGCACT-3’, 5’- GAGTCTGCGAAAGGACTTGG-3’
- GILZ: 5’-TCAATGAGGGCATCTGCAACCG-3’, 5’-CATCAGGTGGTTCTTCACGAGG-3’

### GST Pull-Down Assay

cDNA clone for human alpha isoform of Hsp90 was obtained from the IMAGE consortium. Open reading frame was PCR amplified with gene-specific forward (5´-GAATTCATGCCT GAGGAAACCCAG-3´) and reverse (5´-TCTGAGTTAGTCTACTTCTTCCAT-3´) primers containing EcoRI and XhoI restriction sites overhangs, subcloned into pGEX6 vector (GE Healthcare) and sequence verified. Plasmids were transformed into One Shot BL21 Star (DE3) Chemically Competent E. coli according to the manufacturer’s protocol. Protein expression was induced by the addition of 1 mM isopropyl-β-D-thiogalactoside (IPTG) and GST and GST-Hsp90 were isolated using glutathione-sepharose beads (GE Healthcare). Brain extract (400 µg protein) was prepared from WT or *Fkbp5*^*TPRmut*^ animals by homogenization of total brain in the PBS buffer, pH 7.0, 0.5% NP-40 and protease/phosphatase inhibitor cocktail (Sigma Aldrich). Beads with attached GST or GST-Hsp90 were incubated with brain extracts for 2 h, 4 °C, followed by three times wash with PBS. Proteins were eluted with 10 mM free glutathione, subjected to SDS-PAGE followed by Western blot analysis with anti-FKBP51 specific antibodies.

### Immunoprecipitation

Whole brains from 12-month-old males were lysed by homogenization in cold PBS supplemented with 0.5% NP-40 and complete Protease/Phosphatase Inhibitor cocktail (Sigma). The lysate was cleared by centrifugation (10 min, 25.000 × g, 4 °C). 2 mg of lysate was incubated overnight at 4 °C with the GR affinity resin (sc-393232 AC, Santa Cruz Biotechnology), anti-AR agarose affinity resin (sc-7305 AC, Santa Cruz Biotechnology) or with empty agarose beads (Sigma Aldrich) respectively. The next day, the beads were washed 3 times with Lysis Buffer without detergent and samples were eluted with 0.1 M acetic acid for 10 min and neutralized with concentrated NaOH. One-tenth of the brain lysates and immunoprecipitates were separated by SDS-PAGE, blotted to nitrocellulose membrane (Millipore) and probed with respective antibodies for GR (D6H2L, Cell Signaling), AR (sc-7305, Santa Cruz Biotechnology), Hsp90 (37-9400, Invitrogen), FKBP51 (GTX66429, GeneTex), FKBP52 (sc-100758, Santa Cruz Biotechnology), PP5 (sc-271816, Santa Cruz Biotechnology). Signals were developed using IRDye 800CW donkey anti-rabbit IgG or IRDye 680RD anti-mouse IgG (Li-Cor Biosciences) and quantified by Odyssey imaging system (Li-Cor Biosciences).

### Computational Prediction of Binding Affinity (ΔG) and Dissociation Constant (K_d_)

Prediction of binding affinity (ΔG) and dissociation constant (K_d_) of the wild type and mutated (K352A and R356A) FKBP51 complexed with HSP90 MEEVD peptide was performed using Prodigy webserver (https://wenmr.science.uu.nl/prodigy/) [45, 46]. Since the 3D structure of mouse FKBP51 complexed with MEEVD peptide was not available, we used human FKBP51-MEEVD complex (PDB ID: 5NJX) as a surrogate because of its high similarity and conserveness. Briefly, the structure was downloaded from the PDB server and mutations were introduced and both WT and mutated complex were subjected to a short molecular dynamics simulation to relax the structures using earlier reported protocol [21]. The last frame from each molecular dynamics trajectory was then subjected to calculation of binding affinity (ΔG) and dissociation constant (K_d_) using Prodigy webserver.

### Assessment of Behavioral Phenotype

Behavioral phenotype of *Fkbp5*^*TPRmut*^ male and female mice was assessed using a battery of tests performed at 2 and 12 months of age. Tests were performed in the order from the least to the most stressful protocol (Supplementary Fig. 2). All tests were run between 9:00 and 17:00 in the light phase. The male and female mice were subjected to behavioral tests separately, either on another day or in another experimental room. Before each test, mice were acclimated to the new environment for 1 h. Each apparatus was thoroughly cleaned with water and 70% ethanol after each mouse to remove scent traces.

*General locomotor and exploratory behaviors* were assessed using open field test. Mice were placed in ActiMot2 apparatus (45 cm × 45 cm, 40 cm height, light intensity 80 lx, TSE Systems, 302,020 series) and allowed to explore for 30 min. The apparatus is equipped with a set of evenly spaced infrared light beams and detectors in X and Y axes on two planes. Animal position and movement was determined based on the beam breaks. Vertical activity (rearing) was detected by registering beam breaks on the upper plane.

*Light-dark test* was performed similarly to open field, using ActiMot2 apparatus (TSE Systems, 302,020 series) additionally equipped with a black box dividing the area into two compartments with equal size: light (450 lx) and dark (0 lx), connected with an open door. Mice were placed into the dark side, facing away from the open door, and allowed to move freely between two compartments for 10 min. Time spent in the light compartment was used as a parameter negatively correlated to anxiety.

*Elevated plus maze* was performed using the apparatus consisting of two open arms (35 cm × 6 cm, 0.2 cm wall height) and two closed arms (35 cm × 6 cm, 15 cm wall height) connected by a central platform (6 cm × 6 cm, 0.2 cm wall height). The maze was set 60 cm above the floor. At the beginning of experiment, mice were gently placed at the center platform (100 lx) facing the same close arm and allowed to explore for 5 min. Animal position and movement were tracked using infrared camera located above the center of the maze and analyzed using EthoVision XT 16 software (Noldus). Time spent in open arms was used to assess the anxiety.

*The Y-maze* spontaneous alternation test was used to assess spatial working memory. Apparatus consisted of three identical arms (35 × 6 cm, 15 cm wall height) placed at 120 degrees angle and connected to a center platform (100 lx). Arms were virtually labeled A, B, and C. At the beginning of the experiment mouse was placed at the end of one arm with nose facing the center and allowed to explore for 5 min. Starting arms were assigned for animals in a pseudo-random manner, so equal number of mice from each group started from each arm. Mice were recorded using a camera located above the center of the maze. The videos were manually analyzed by two researchers blind to the animal group. Order of arm entries was recorded and triplets of the respective alterations were counted: spontaneous alternations (e.g., ABC, BCA, CBA), alternate arm returns (e.g., ABA, BAB, CBC), same arm returns (e.g., ABB, BBC, CCA) [47]. The percentage of spontaneous alternations, calculated as the number of spontaneous alternations divided by the total number of triplets, was used as an index of spatial working memory.

*Novel object recognition* (NOR) memory was assessed using a paradigm where mice were exposed to sets of different objects. The apparatus consisted of four arenas (45 cm × 45 cm, 40 cm height) and a set of objects differing in shape, color, and texture (plastic red oval, white plastic glue bottle, clear plastic cell culture flask filled with gravel). The illumination level at each arena centre was adjusted to around 20 lx. Before the proper experiment mice were allowed to explore empty arenas for 10 min on two consecutive days. During the first session mice were allowed to explore a pair of identical objects for 5 min. After 30 min, short-term memory was assessed: one familiar object was replaced with a novel object and mice were allowed to explore for 5 min. 24 h after the first session, long-term memory was assessed by allowing mice to explore a set of one familiar and another new object. The location of familiar object was swapped for each mouse between short-term and long-term memory assessment to eliminate place preference bias. The type of objects and their positions were balanced among the groups to eliminate object preference bias. The experiment was recorded using a camera located above the center of the apparatus. The time spent in exploring each object was manually scored by researcher blind to the group. The discrimination index was calculated as the time spent exploring the novel object divided by total exploration time on both objects during the session. Exploration was considered when a mouse was facing the object and paying attention to it and the distance between the object and the nose was below 2 cm. Chewing, sitting, climbing, and leaning on the objects was not considered as exploration.

*Fear conditioning test*. Negative stimuli-based memory was assessed using a 3-day fear conditioning paradigm using the TSE Multi Conditioning System (256,060 series). On day 1 (conditioning), mice were placed in rectangular arenas (20 cm × 20 cm, 40 cm height) made of plexiglass with one transparent wall and the other three black and non-transparent walls, with grid floor and equipped with a set of infrared sensors on two planes to detect animal position and movement. Arenas were in soundproof chambers. After an initial phase of habituation (2 min) during which mice were allowed to freely explore the arena, a sound (55 dB sound at 5000 Hz) was applied for 30 s. Immediately after the sound mice received a foot shock (0.3 mA) for 2 s. The sound-shock cycle was repeated 3 times with a 50-second interval. On day 2 (context-based memory test), mice were placed in the same arena setup for 3 min and allowed to explore without any sound or foot shock. Freezing during the habituation phase of day 1 and during the day 2 test were detected automatically and compared to indicate the context-based memory of aversive stimulus. On day 3 (cue-based memory test), mice were placed in the apparatus equipped with changed arena setup. The new arenas were of cylindrical shape (20 cm diameter × 40 cm height), totally transparent, with smooth floor. To completely change the context from the previous phases, during this day arenas were cleaned with a water solution of hypochlorous acid instead of 70% alcohol. After 2 min of free exploration, the same sound (55 dB sound at 5000 Hz) as during the day 1 was played for 2 min continuously. Freezing before and during the sound was used as an index of cue-based memory of aversive stimulus.

*Forced swimming test* assessment of despair. Mice were placed in plexiglass cylinders (17 cm diameter × 25 cm height) filled with clean water (23–25 °C, 15 cm water depth) for 6 min and recorded from the side with a digital camera. White untransparent dividers were put between cylinders to prevent mice from seeing each other during the test. Their immobility time was manually scored during the last 4 min by two researchers blind to the group.

*The Rotarod test* was used for assessment of motor coordination. The apparatus (Model 47,600, Ugo Basile) consisted of a rotating horizontal bar (3 cm diameter, 15 cm above the instrument floor) with ridges allowing for the grip. On the first day mice were habituated to the apparatus by placing them on the bar revolving with a fixed speed of 4 RPM for 90 s two times with a 40-min interval. During the following three consecutive days, each mouse obtained 3 training sessions daily with a 40-min interval. In each session, mice were placed on the bar at a speed of 4 RPM which accelerated constantly to 40 RPM over 5 min. A session ended when the mouse fell off the bar or started to perform passive rotations by gripping the ridges tightly or maintained its balance for maximum 5 min. Latency to fall was automatically recorded by the apparatus.

### Assessment of Endocrine Phenotype After Acute Stress

At 3 months of age, WT and *Fkbp5*^*TPRmut*^ male and female mice were restrained using 50 mL Falcon tubes with additional holes to allow for ventilation for 15 min [48]. After 30-min recovery, blood was collected from the saphenous vein and centrifuged at 1500 *g* for 10 min to isolate plasma. Plasma levels of CORT and ACTH were detected by CORT Competitive ELISA Kit (Cat. No. EIACORT, Invitrogen) and ACTH ELISA Kit (Cat. No. ab263880, Abcam), according to manufacturer’s instructions.

### Assessment of Metabolic Phenotype

At 2 months of age, metabolic phenotype of *Fkbp5*^*TPRmut*^ male and female mice was assessed under CD (10% kcal from fat, D12450J) or HFD (60% kcal from fat, D12492, Research Diets, New Brunswick, NJ, USA) for 5 continuous weeks. Body weight was measured once a week throughout the experiment. During the last week, the whole-body fat mass and lean mass was measured by EchoMRI-100™ and GTT was performed. In GTT, after 5-h fasting mice were i.p. injected with 20% sterile glucose solution in saline at 1.5 g/kg fat-free mass. Blood samples were collected at the distal tail vein repeatedly at 0 (fasting glucose level), 15, 30, 60, 90 and 120 min after glucose load. Blood glucose levels were measured by glucometer (Contour XT).

### Statistical Analysis

Data are displayed as mean ± standard error of the mean (SEM). GraphPad Prism software (version 9.3.1) were applied to draw graphs and calculate the statistical significance. *p* < 0.05 was considered statistically significant. Shapiro-Wilk method was used to test data normality. Statistical significance was determined by using Unpaired Student’s *t* test (two-tailed), Mann-Whitney *U* test (two-tailed) or two-way analysis of variance (ANOVA) followed by Sidak’s multiple comparison test.

### Supplementary Information

ESM 1 (DOCX 743 KB)

## Data Availability

The source data and statistical analyses underlying all figures are provided as Source Data files. The datasets generated during and/or analyzed during the current study are available from the corresponding author upon reasonable request.
