# Supplementary material for: FKBP51-Hsp90 Interaction-Deficient Mice Exhibit Altered Endocrine Stress Response and Sex Differences Under High-Fat Diet
Source: Mol Neurobiol. 2023 Sep 19;61(3):1479–94. doi: 10.1007/s12035-023-03627-x (PMC10896785; doi:10.1007/s12035-023-03627-x)
Supplement: Supplementary file 1 — (DOCX 743 KB) [file 12035_2023_3627_MOESM1_ESM.docx]

**FKBP51-Hsp90 Interaction-Deficient Mice Exhibit Altered Endocrine Stress Response and Sex Differences Under High-Fat Diet**

**Supplementary Materials**

Lisha Wang^1*^, Jakub Wojcieszak^1,2*^, Rajnish Kumar^3^, Zhe Zhao^1,4^, Xuelian Sun^1,5^, Shaoxun Xie^1^, Bengt Winblad^1,6^, Pavel F. Pavlov^1^

*^1^Department of Neurobiology, Care Sciences and Society, Division of Neurogeriatrics, Karolinska Institutet, 17164 Solna, Sweden;*

*^2^Department of Pharmacodynamics, Medical University of Lodz, 90151 Lodz, Poland;*

*^3^Department of Pharmaceutical Engineering & Technology, Indian Institute of Technology (BHU), 221005 Varanasi, India;*

*^4^Department of Toxicology, School of Public Health, Peking University, 100191 Beijing, China;*

*^5^National Clinical Research Center for Geriatrics and Department of Gerontology and Geriatrics, West China Hospital, Sichuan University, 610041 Chengdu, China*;

*^6^Theme Inflammation and Aging, Karolinska University Hospital, 14186 Huddinge, Sweden.*

*^*^ These authors contributed equally to this work*

Corresponding author:

Pavel F. Pavlov, E-mail: [pavel.pavlov@ki.se](mailto:pavel.pavlov@ki.se)

**Supplementary Table 1.** Prediction of binding affinity (**ΔG**) and dissociation constant (**K_d_**) of the wild-type human FKBP51 and K352A and R356A mutated human FKBP51 complexed with Hsp90 MEEVD peptide.

| **Protein-protein complex** | **ΔG (kcal mol^-1^)** | **K_d_(M) at** 25 **℃** | **^#^ICs charged-charged** | **^#^ICs charged-polar** | **^#^ICs charged-apolar** | **^#^ICs polar-polar** | **^#^ICs polar-apolar** | **^#^ICs apolar-apolar** |
| --- | --- | --- | --- | --- | --- | --- | --- | --- |
| 5NJX | -6.9 | 8.8 e^-06^ | 9 | 3 | 12 | 0 | 5 | 7 |
| 5NJX  Mutated | -5.7 | 7.0 e^-05^ | 5 | 4 | 7 | 0 | 4 | 6 |

**^#^**Number of intermolecular contacts (ICs) at the interface within the threshold distance of 5.5 Å.


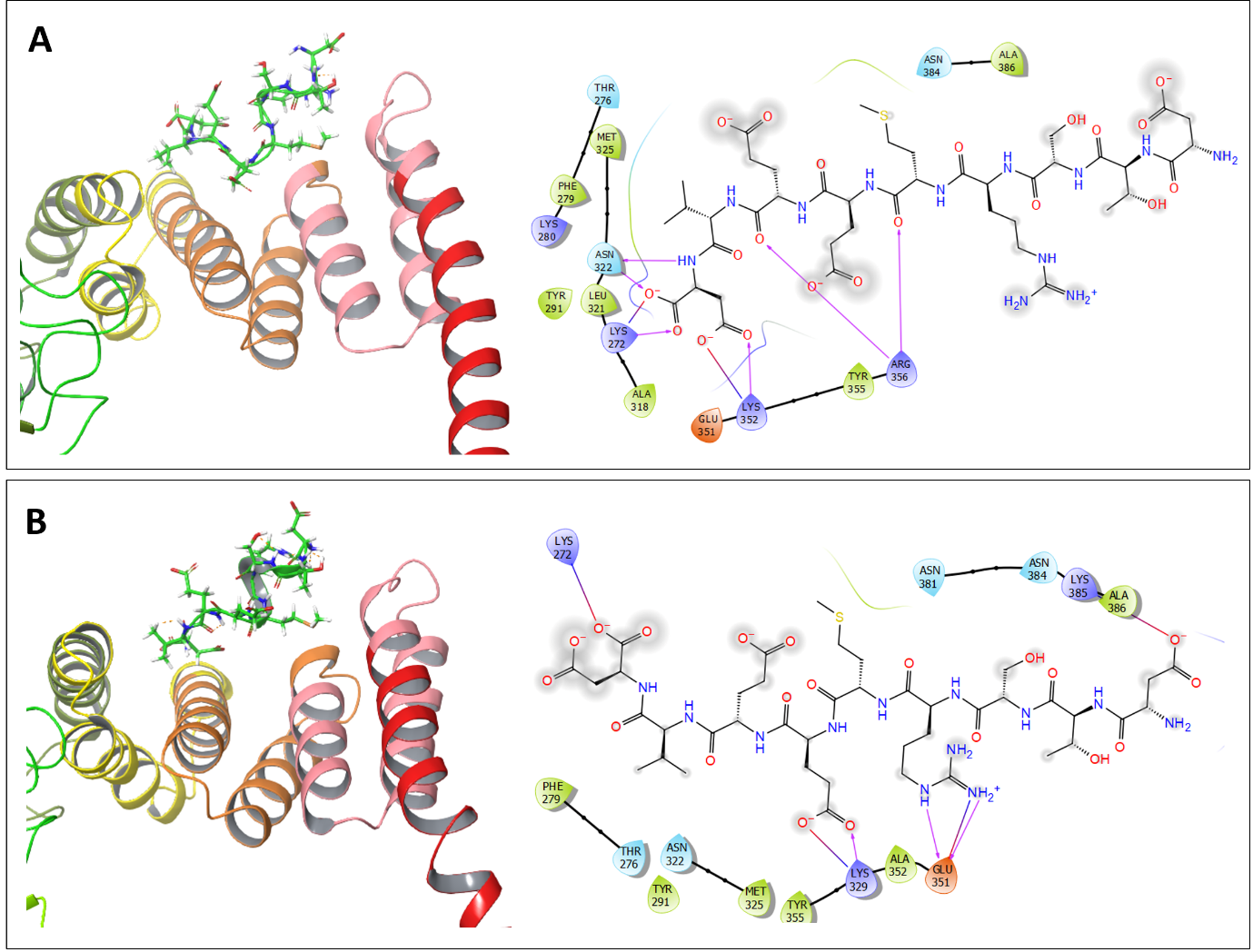


**Supplementary Fig. 1.** Interaction of Hsp90 MEEVD peptide with the TPR domain of wild type human FKBP51 (**A**) and K352A and R356A mutated human FKBP51 (**B**). The two-dimensional interaction plot for the wild type FKBP51 (**A**) shows “dicarboxylate clamp” responsible for the anchoring of MEEVD peptide to the TPR domain while the mutated FKBP51 (**B**) shows loss of the formation of dicarboxylate clamp leading to disruption of FKBP51 binding to the MEEVD peptide.


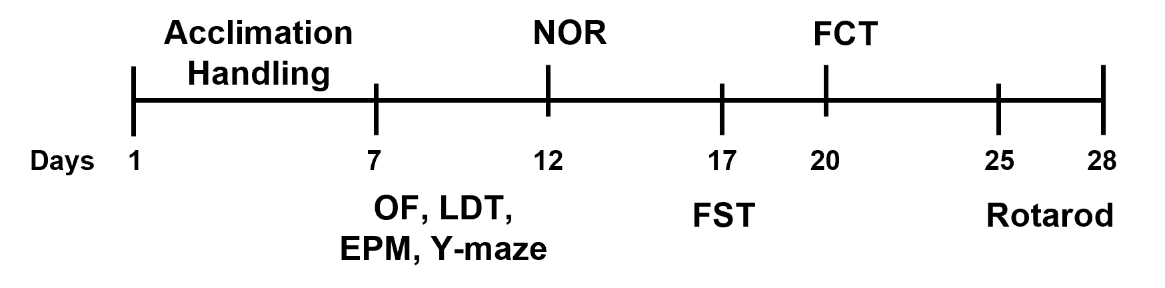


**Supplementary Fig. 2.** Schematic overview of behavioral tests for *Fkbp5^TPRmut^* male and female mice at 2 or 12 months of age. After acclimation, mice were exposed to open field (OF), light-dark test (LDT), elevated plus maze (EPM), Y-maze, novel object recognition (NOR), forced swimming test (FST), fear conditioning test (FCT) and rotarod test. Between each two tests, mice were left to recover for 1-3 days according to the stress level.
